# Supplementary material for: Solid Sampling Pyrolysis Adsorption-Desorption Thermal Conductivity Method for Rapid and Simultaneous Detection of N and S in Seafood
Source: Molecules. 2022 Dec 15;27(24):8909. doi: 10.3390/molecules27248909 (PMC9786581; doi:10.3390/molecules27248909)
Supplement: Supplementary file 1 [file molecules-27-08909-s001.zip › molecules-2092112-supplementary.pdf]

Supporting information for

Rapid and simultaneous detection of N and S in seafood using solid sampling pyrolysis adsorption-desorption thermal conductivity method

Derong Shang[1], Wenyan Gu[1], Yuxiu Zhai[1], Xiaofeng Sheng[1], Yanfang Zhao[1], Jinsong Ning[1*], Xuefei Mao[2*], Haiyan Ding[1], and Xuming Kang[1]

[1]Key Laboratory of Testing and Evaluation for Aquatic Product Safety and Quality, Ministry of Agriculture and Rural Affairs, China; Yellow Sea Fisheries Research Institute, Chinese Academy of Fishery Sciences, Qingdao 266071, China

[2]Institute of Quality Standard and Testing Technology for Agro-products, Chinese Academy of Agricultural Sciences, and Key Laboratory of Agro-food Safety and Quality, Ministry of Agriculture and Rural Affairs, Bei-jing 100081, China

Corresponding authors: Tel: +86 13906420075, Fax: +86-532-85825917.

E-mail address: ningjs@ysfri.ac.cn (J. S. Ning); mxf08@163.com or maoxuefei@caas.cn (X. F. Mao)

Table S1. Results of N recovery rate in live Fenneropenaeus chinensis flesh

sample number	Content of N (g/100g)	Average Content of N (g/100g)	Spiked content (g/100g)	detection result (g/100g)	recovery rate（%）
1	3.20	3.21	4.83	4.72	97.72
2	3.22		4.83	4.71	97.52
3	3.22		4.83	4.70	97.31
4	3.21		6.45	6.46	100.2
5	3.22		6.45	6.37	98.76
6	3.20		6.45	6.38	98.91

Table S2. Results of S recovery rate in live Fenneropenaeus chinensis flesh

sample number	Content of S (g/100g)	Average Content of S (g/100g)	Spiked content (g/100g)	detection result (g/100g)	recovery rate （%）
1	0.0074	0.0070	0.163	0.172	105.5
2	0.0071		0.163	0.158	96.93
3	0.0068		0.163	0.170	104.3
4	0.0066		0.319	0.326	102.2
5	0.0069		0.319	0.335	105.0
6	0.0073		0.319	0.316	99.06

Table S3. Results of recovery rate of N content in ground fish powder

sample number	Content of N (g/100g)	Average Content of N (g/100g)	Spiked content (g/100g)	detection result (g/100g)	recovery rate（%）
1	5.01	5.02	7.51	7.18	95.61
2	5.00		7.51	7.16	95.34
3	5.02		7.51	7.20	95.87
4	5.02		10.01	10.10	100.9
5	5.04		10.01	10.14	101.3
6	5.03		10.01	10.12	101.1

Table S4. Results of recovery rate of S content in ground fish powder

sample number	Content of S (g/100g)	Average Content of S (g/100g)	Spiked content (g/100g)	detection result (g/100g)	recovery rate (%)
1	0.0417	0.041	0.323	0.298	92.26
2	0.0406		0.323	0.323	100.0
3	0.0411		0.323	0.318	98.45
4	0.0412		0.605	0.606	100.2
5	0.0414		0.605	0.574	94.88
6	0.0405		0.605	0.595	98.35

Table S5. Comparison between this method and national standard methods for N and S content detection in many seafood species (n =30)

Sample	This method		GB 5009.5-2016	GB 5009.34-2016
	Content of N (%)	Content of S (g/kg)	Content of N (%)	Content of S (g/kg)
Sea cucumber	**6.86**	**<0.01**	**6.88**	**<0.01**
Dried shrimps	**6.36**	**0.02**	**6.33**	**0.02**
Grilled shredded squid	**4.91**	**<0.01**	**4.94**	**<0.01**
Grilled sea fish fillet	**6.55**	**<0.01**	**6.51**	**<0.01**
Roast cod fillets	**5.65**	**0.04**	**5.67**	**0.04**
Scallops	**6.72**	**ND**	**6.76**	**ND**
Roasted shrimp	**8.93**	**0.18**	**8.91**	**0.16**
Dried silver carp	**6.16**	**<0.01**	**6.18**	**<0.01**
Grilled fish slices	**5.04**	**0.04**	**5.02**	**0.03**
Japanese sea cucumber	**5.85**	**<0.01**	**5.88**	**<0.01**
Dalian sea cucumber	**7.42**	**<0.01**	**7.45**	**<0.01**
shrimp skin	**7.39**	**ND**	**7.28**	**ND**

Sample	This method		GB 5009.5-2016	GB 5009.34-2016
	Content of N (%)	Content of S (g/kg)	Content of N (%)	Content of S (g/kg)
shrimp skin	**6.30**	**ND**	**6.36**	**ND**
hrimp skin	**6.82**	**ND**	**6.85**	**ND**
squid silk	4.29	**ND**	4.26	**ND**
grilled cod roe	5.17	**ND**	5.19	**ND**
squid litter	5.20	**ND**	5.24	**ND**
grilled Hippo fish	6.09	**ND**	6.05	**ND**
dried yellow croaker	8.38	**ND**	8.32	**ND**
Moringa fish	6.06	0.015	6.02	0.012
scallop drying	7.26	0.016	7.22	0.012
sea M	9.31	<0.01	9.36	<0.01
hemizygous fish	6.69	0.018	6.65	0.015
shrimp skin	4.59	0.069	4.47	0.066
smoked Spanish fish	4.71	**ND**	4.77	**ND**
dried pompano	5.90	0.014	5.94	0.012
fishstem	5.68	0.018	5.65	0.016
shortening crab	4.26	0.022	4.22	0.024
clam stem	5.91	0.021	5.87	0.018
eel trunk	6.02	0.014	6.04	0.013
Octopus trunk	4.18	<0.01	4.24	<0.01
dried carrier fish	5.07	<0.01	5.02	<0.01
tap fish dry	5.84	<0.01	5.81	<0.01
sea M	9.12	<0.01	9.14	<0.01
**Turbot**	**2.65**	/	**2.69**	/
**Snakehead**	**3.62**	/	**3.65**	/
**Mandarin fish**	**3.11**	/	**3.12**	/

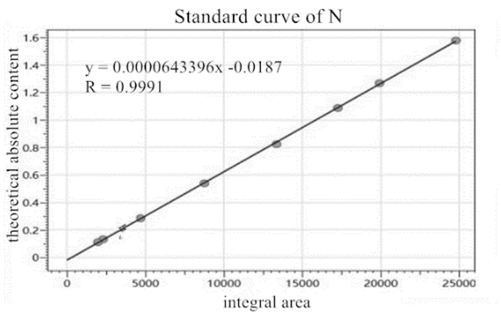

Figure S1. Standard curve of N ranging from 2.24mg to 31.61mg. A group of nori CRM (GBW10023) (5.0 ± 0.3%) were measured under 900°C pyrolysis temperature, and other parameters are performed according to Table 1.

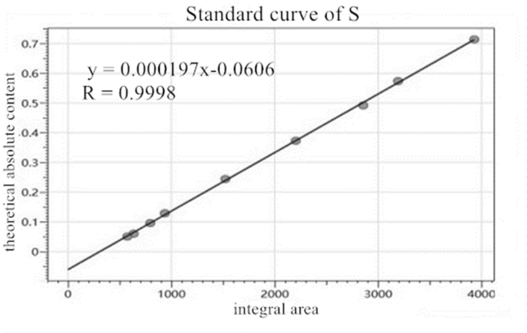

Figure S2. Standard curve of S ranging from 2.24mg to 31.61mg. A group of nori CRM (GBW10023) (2.26 ± 0.14%) were measured under 900°C pyrolysis temperature, and other parameters are performed according to Table 1.

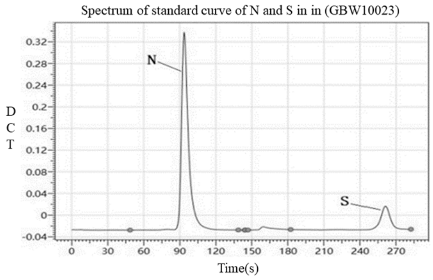

Figure S3. Spectrum of N and S in a nori CRM sample (GBW10023)

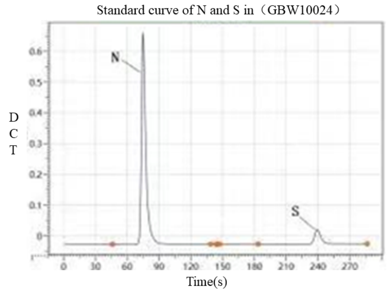

Figure S4. Spectrum of N and S in Standard substance Pectinidae（GBW10024）

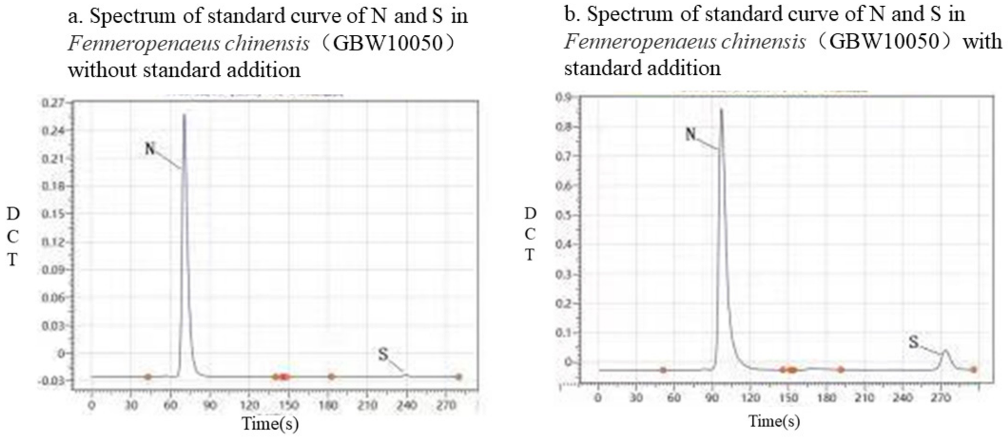

Figure S5. Spectrum of N and S of *Fenneropenaeus chinensis* (GBW10050). a: Spectrum of N and S of *Fenneropenaeus chinensis* (GBW10050) without standard addition; b: Spectrum of N and S of *Fenneropenaeus chinensis* (GBW10050) with standard addition.

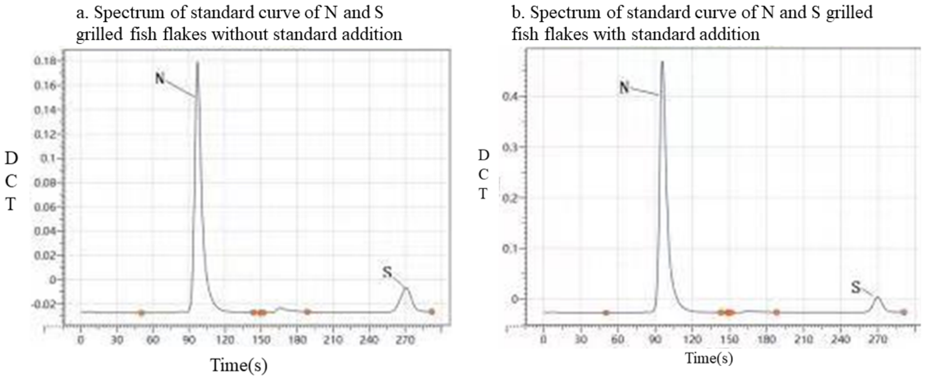

Figure S6. Spectrum of N and S in grilled fish flakes. a: Spectrum of N and S in grilled fish flakes without standard addition; b: Spectrum of N and S in spiked grilled fish flakes with standard addition.
